# Safety and efficacy of a novel oral chewable combination tablet containing sarolaner, moxidectin and pyrantel (Simparica Trio™) against natural flea infestations in client-owned dogs in the USA

**DOI:** 10.1186/s13071-020-3952-3

**Published:** 2020-03-01

**Authors:** Kristina Kryda, Sean P. Mahabir, Tammy Inskeep, Jady Rugg

**Affiliations:** Zoetis, Veterinary Medicine Research and Development, 333 Portage St., Kalamazoo, MI 49007 USA

**Keywords:** Clinical field study, *Ctenocephalides felis felis*, FAD, Flea allergy dermatitis

## Abstract

**Background:**

One randomized, controlled clinical field study was conducted in 18 general veterinary practices throughout the USA to evaluate the safety and efficacy of a novel oral chewable combination tablet, Simparica Trio™, containing sarolaner, moxidectin and pyrantel for the treatment and prevention of fleas on dogs.

**Methods:**

Client-owned dogs, from households of three or fewer dogs were eligible for enrollment. Four hundred and twenty-two dogs from 251 households were enrolled. Households were randomly assigned in a 2:1 ratio to treatment with either Simparica Trio™ at the minimum label dose of 1.2 mg/kg sarolaner, 24 µg/kg moxidectin and 5 mg/kg pyrantel (as pamoate salt) or afoxolaner (NexGard^®^, Boehringer-Ingelheim) at the label dose. One dog per household was selected as the primary dog for efficacy evaluations. Treatments were dispensed and dogs were dosed in their home environment on Day 0 and on approximately Day 30. Flea counts and examination for clinical signs of flea allergy dermatitis (FAD) were performed at the initial visit the day before or on Day 0 prior to treatment and on Days 30 and 60. Additionally, all dogs were examined for general health at each visit and blood and urine were collected for clinical pathology at screening and Day 60.

**Results:**

Simparica Trio™ reduced geometric mean live flea counts by 99.0% by Day 30 and by 99.7% by Day 60. As a result of the rapid reduction in flea infestations, clinical signs associated with FAD substantially improved following treatment. Simparica Trio™ was well-tolerated and a diverse range of concomitant medications were administered to dogs during the course of the study. Simparica Trio™ chewable tablets were well-accepted by dogs, with the majority of flavored chewable tablets (91.9%) voluntarily consumed by free choice without, or when offered in food.

**Conclusions:**

Simparica Trio™ administered orally once monthly for two consecutive treatments was safe and effective against natural flea infestations and substantially improved clinical signs associated with FAD in client-owned dogs in a field study conducted in the USA.

## Background

Fleas are major ectoparasites of dogs and cats globally and the cat flea, *Ctenocephalides felis felis* (Bouché, 1835) (Siphonaptera: Pulicidae), is the most common [1, 2]. Fleas cause local irritation due to their blood-feeding and heavy infestations, especially in young or debilitated animals, can lead to anemia [3]. The clinical signs associated with flea feeding, also known as flea bite dermatitis, include pruritus, erythema, papules, scaling, alopecia and dermatitis/pyodermatitis, and are generally transient and resolve rapidly when the fleas are controlled. However, exposure to fleas may lead to the development of flea allergy dermatitis (FAD) which is the most common dermatologic disease of domestic dogs [4]. Once a dog is sensitized, recurrence of clinical signs can be initiated by a few bites, although the threshold of sensitivity varies between individual dogs [5, 6]. Successful management of FAD depends on eliminating fleas as they provide the allergenic challenge, and continuous prevention of flea infestations is recommended [7]. Fleas transmit several pathogens, including agents of zoonotic diseases, such as *Rickettsia felis* [8], *Rickettsia typhi* [9] and *Bartonella henselae* [10, 11], and are intermediate hosts for the dog tapeworm [2]. In the absence of their primary hosts, cat fleas will readily feed on humans [3, 12]. Effective flea control is thus an important consideration for pet owners and veterinarians given the ubiquitous nature of fleas, blood-feeding habits, and possibility of transmitting diseases to the dog including zoonotic disease agents [13]. To prevent the negative effects and irritation caused by flea feeding and reduce the risks of disease transmission, year-round flea control should be considered for pets in most geographical areas [1, 2]. The effective prevention and control of fleas relies on parasiticides with fast onset of action and consistent efficacy over the dosing interval, as well as pet owner compliance with treatment recommendations.

Besides fleas, dogs are afflicted with a number of other external and internal parasites which have deleterious effects on their hosts and can potentially transmit pathogens to both dogs and humans. Ticks cause direct irritation through their blood feeding activity and heavy infestations may cause anemia and even death; also ticks may transmit disease organisms that can cause severe, even life-threatening illnesses in both dogs and humans [14]. Heartworm is a filariid nematode transmitted by mosquitoes that causes severe disease and potentially death in infected dogs. Gastrointestinal parasites such as roundworms and hookworms commonly infect dogs and are potentially zoonotic. Year-round preventative treatment of most, if not all, of these common parasites of dogs with broad-spectrum parasiticides is recommended in the USA to ensure effective management of parasites and to reduce the risk of the diseases they can transmit [15].

Recently, a chewable combination oral formulation including sarolaner, moxidectin and pyrantel (Simparica Trio™, Zoetis, Parsipanny, NJ, USA) has been developed that provides prevention of heartworm and lungworm disease, treatment and control from flea and tick infestations for 1 month and treatment of roundworm and hookworm infections in dogs. One clinical field study was conducted to evaluate the palatability, safety and efficacy of the chewable combination tablet (Simparica Trio™) administered orally for 2 months per label for the treatment and prevention of natural flea infestations in dogs presented as veterinary patients in the USA.

## Methods

The study was conducted using client-owned dogs enrolled from 18 clinics in various regions of the USA. The study design was a single-masked, randomized, multi-center clinical trial using NexGard^®^ (Boehringer-Ingelheim, Ingelheim, Germany) as a positive control. The study complied with Good Clinical Practice guidelines [16] and was conducted in accordance with the World Association for the Advancement of Veterinary Parasitology (WAAVP) guidelines for evaluating the efficacy of parasiticides for the treatment, prevention and control of flea and tick infestation on dogs and cats [17].

### Animals

The patient population was enrolled from dogs presenting to veterinary clinics from a diverse range of households and living conditions representing the range of typical clients for North America. Only one dog from a household could be included as a primary patient for evaluation of efficacy, and households with additional dogs (up to a total of three dogs) and/or cats could be enrolled. To be included in the study, at least one dog in the household had to harbor at least 10 fleas. There were no breed or sex restrictions; however, dogs had to be at least 8 weeks of age and weigh at least 1.8 kg. Dogs that were pregnant, lactating or intended for breeding were not eligible for enrollment. Dogs had to be amenable to handling for the study activities and owners had to be able to administer oral medications. Dogs with stabilized pre-existing conditions under veterinary care and who were expected to survive the duration of the study could be included, but dogs with existing unstable medical conditions that might confound the study were excluded. Dogs could not be included if they had been treated with a flea preventative or injectable moxidectin (ProHeart 6^®^, Zoetis, Parsippany, NJ, USA) within its labeled “protective period” at the start of the study, i.e., six months for ProHeart 6^®^, 1 month for most other products such as Advantage Multi^®^, Frontline^®^, NexGard^®^ or longer such as 12 weeks for Bravecto^®^. Additionally, dogs could not be included if they were older than 6 months of age and tested positive for heartworm infection.

Dogs were kept under their normal home conditions for the duration of the study. Other than the experimental treatments, dogs in the household were not allowed to use products that had activity against fleas (systemic and/or over-the-counter treatments including insecticidal shampoos or collars). Owners were encouraged to treat any cats that shared the same environment with a suitable commercially available product but any environmental or premise flea treatments were prohibited for the study duration. Non-insecticidal shampoos were permitted to be used; however, primary dogs could not be bathed within 3 days prior to a flea assessment. Dogs were allowed to receive corticosteroids during the study, however dogs that used these medications were excluded from the skin assessment analysis.

### Design

The study utilized a randomized complete block design within clinic. As dogs presented to the clinic and were determined to be eligible for enrollment, their household was allocated randomly to treatment with the combination product or with afoxolaner in the ratio of 2:1. If a single dog in a household presented with 10 or more fleas, this animal was selected as the primary dog. When more than one dog in a household met this criterion, the dog whose first letter of their name came alphabetically first was selected as the primary dog. All other dogs were designated as supplementary dogs. Primary dogs received both efficacy and safety evaluations. All the other dogs in a household received the same treatment as the primary dog but were included in safety evaluations only. All dogs were included in the palatability assessments.

At the initial screening visit, all dogs in a household were weighed, given a physical exam, had blood collected for hematology and blood chemistry and had urine collected for urinalysis. Blood was also collected for adult heartworm (antigen and microfilaria) testing if the dog was 6 months of age or older. Flea counts were conducted by clinic personnel trained to a standardized methodology. The dogs were systematically combed using a commercial fine-tooth flea comb to remove and count fleas, initially while standing starting from the head, then proceeding caudally along the dorsum. The dog was then placed on each side and then on its back for combing of the sides and ventral surfaces. Dogs were repeatedly combed for a minimum of 10 min, and if live fleas were recovered in the final 2 min, combing was continued in 2-min increments until no fleas were recovered within a 2-min period. Fleas maintaining an upright orientation or moving in a coordinated manner were considered to be live. Only live flea counts were recorded, and species identification was not performed on these counted fleas.

Once the primary dog was identified, it was assessed by the veterinarian for the clinical signs associated with FAD. The severity of pruritus, papules, erythema, scaling, alopecia and dermatitis/pyodermatitis was assessed as: absent (no observable abnormalities); mild (intensity/density of the abnormality was low and only a small area of the dog’s body was affected); moderate (the abnormality was of great intensity/density over a small area or was of lesser intensity/density but affected a large area of the dog’s body); or severe (the abnormality was of great intensity/density and covered a large area of the animal’s body). Personnel performing the flea counts, skin assessments, or other observations were masked to treatment allocation.

The chewable combination tablets were provided in six different tablet strengths to provide dose ranges of 1.2–2.4 mg/kg sarolaner, 24–48 µg/kg moxidectin and 5–10 mg/kg pyrantel (as pamoate salt). Commercial afoxolaner tablets were dosed according to the commercial product label directions to provide doses of 2.5–6.3 mg/kg afoxolaner. Owners were provided with the treatments and instructed on treatment and palatability assessment methods at the clinic, and then administered the tablets and evaluated product consumption at home for all dogs in the household on that or the following day. Day 0 was defined as the day on which the primary dog received its first dose. The dose could be offered at any time of the day, with or without food. To assess palatability, owners were instructed to first offer the tablet(s) without food. If the tablet(s) were not consumed within 5 min, then they were to be offered in a small amount of food. If the tablet(s) were not consumed with food, then they were to be given by “pilling” (placing the tablet(s) at the back of the mouth and gently holding the mouth shut until the dog swallowed). If a tablet was broken during chewing or if pieces of a tablet fell from the dog’s mouth during chewing, the owner was to recover and re-offer or re-dose the tablet or pieces of the tablet. Unmasked study personnel from the clinic contacted the owner within 2 days of dispensing to ensure the treatment was administered successfully and determine if any adverse events had been noted.

All dogs were presented to the clinics on Days 30 and 60 with a target visit window of ± 3 days. At each visit, primary dogs had flea counts and assessments for the signs of FAD performed and all dogs were weighed and examined for general health. On Day 30, owners were dispensed the appropriate tablets to be dosed in the home environment as described above. On Day 60, all dogs had blood and urine collected for hematology, blood chemistry and urinalysis. Any dogs that presented for an unscheduled visit during the study were examined by a veterinarian for any abnormal health issues.

### Statistical analysis

To be included in the efficacy assessment, a clinic had to enroll at least 2 primary dogs in each treatment group and have at least two primary dogs in each group assessed on Day 30 and Day 60. Paired data for Days 0 and 30 and for Days 0 and 60 for live flea counts were log-transformed [log_e_(count + 1)] and analyzed by treatment group with mixed linear models for repeated measures (SAS version 9.4). The models included the fixed effect of time and the random effects of clinic, animal within clinic, the interaction of clinic, time, and error. Live flea counts were summarized with arithmetic and geometric means by treatment group and timepoint. Geometric means were estimated using the back-transformed least squares means for treatment groups at each time point and used to calculate percent effectiveness using the formula [(C − T) / C] × 100, where C is the pre-treatment mean flea count and T is the post-treatment mean flea count.

To assess the impact of treatment on the clinical signs associated with FAD, any primary dog with at least one of these clinical signs present at the initial screening visit, that did not receive concurrent corticosteroids or any other treatments that could confound skin evaluation, was evaluated at subsequent visits. For each clinical sign, improvement in an individual dog was defined as a reduction of at least one assessment category from the screening visit to the Day 60 visit. The percentages of dogs with improvement were calculated for each treatment group for each clinical sign.

## Results

### Demographics

A total of 422 client-owned dogs from 18 clinics in various regions of the USA were enrolled and included in safety evaluations (Table 1) with 278 dogs receiving the combination product and 144 dogs afoxolaner. Of these, 251 (167 in the combination group and 84 in the afoxolaner group) were primary dogs enrolled for efficacy evaluations. Of the total dogs (*n* = 422), the sex ratio was approximately the same with 210 (49.8%) females and 212 (50.2%) males. There were slightly more neutered animals (72.9%) in the afoxolaner group than the combination product group (63.3%). The median age of enrolled dogs was 5 years for both groups (means of 5.4 and 5.3 years for the combination product group and afoxolaner group respectively, range 8 weeks to 17 years). Breed distribution was similar for both groups, purebred dogs comprised about 50% of the enrolled population with Labrador Retrievers, Dachshunds, Chihuahuas, Shih Tzus, American Pit Bull Terriers, Golden Retrievers and German Shepherd Dogs being enrolled most frequently. Dogs with short hair coats represented 54.0% of the enrolled population, dogs with medium coats 33.6% and dogs with long coats 12.3%. Living conditions for the dogs were similar for both groups; ~ 52% spent time primarily indoors and ~ 43% spent time both indoors and outdoors, the remainder (~ 5%) were primarily outdoors. For primary dogs (*n* = 251), these population characteristics were also similarly represented in the two treatment groups (Table 2). The majority of primary dogs (64.5%) were housed with up to two other dogs and/or up to 31 cats. This household animal pattern was similar for both treatment groups. Thus, the patient demographics (sex, age, breed, coat, home environment etc.) for the study population were similar between the two treatment groups.

**Table 1:** Clinic location and number/percentage of dogs in a clinical field study investigating the safety and efficacy of Simparica Trio™

Clinic location	Primary dogs (efficacy)						All dogs (safety)					
	Simparica Trio™		Afoxolaner		Total		Simparica Trio™		Afoxolaner		Total	
	*n*	%	*n*	%	*n*	%	*n*	%	*n*	%	*n*	%
Lake Worth, FL	10	6.0	5	6.0	15	6.0	13	4.7	6	4.2	19	4.5
Pensacola, FL^a^	1	0.6	0	0.0	1	0.4	2	0.7	0	0	2	0.5
Gainesville, FL	16	9.6	8	9.5	24	9.6	25	9.0	12	8.3	37	8.8
Savannah, GA	20	12.0	10	11.9	30	12.0	33	11.9	15	10.4	48	11.4
Bogart, GA^a^	1	0.6	0	0.0	1	0.4	1	0.4	0	0	1	0.2
Metairie, LA	15	9.0	7	8.3	22	8.8	21	7.6	16	11.1	37	8.8
Wichita Falls, TX	11	6.6	6	7.1	17	6.8	22	7.9	10	6.9	32	7.6
Sequin, TX	6	3.6	3	3.6	9	3.6	7	2.5	7	4.9	14	3.3
Lumberton, TX^a^	0	0	1	1.2	1	0.4	0	0	2	1.4	2	0.5
Bartlesville, OK	18	10.8	9	10.7	27	10.8	38	13.7	16	11.1	54	12.8
San Diego, CA^a^	1	0.6	0	0.0	1	0.4	1	0.4	0	0	1	0.2
Riverside, CA^b^	3	1.8	2	2.4	5	2.0	5	1.8	4	2.8	9	2.1
Farragut, TN	16	9.6	8	9.5	24	9.6	33	11.9	17	11.8	50	11.8
Memphis, TN	10	6.0	5	6.0	15	6.0	11	4.0	6	4.2	17	4.0
Raleigh, NC	9	5.4	5	6.0	14	5.6	13	4.7	6	4.2	19	4.5
Springfield, MO	16	9.6	8	9.5	24	9.6	33	11.9	15	10.4	48	11.4
Quakertown, PA	10	6.0	5	6.0	15	6.0	16	5.8	9	6.3	25	5.9
Caledonia, MI	4	2.4	2	2.4	6	2.4	4	1.4	3	2.1	7	1.7
Total	167	100	84	100	251	100	278	100	144	100	422	100

^a^Site not included in efficacy evaluation as insufficient evaluable cases (2 primary dogs in each treatment) enrolled

^b^Site not included in efficacy evaluation as insufficient evaluable cases (2 primary dogs in each treatment) returned for assessment on Day 30

*Abbreviations*: CA, California; FL, Florida; GA, Georgia; LA, Louisiana; MI, Michigan; MO, Missouri; NC, North Carolina; OK, Oklahoma; PA, Pennsylvania; TN, Tennessee; TX, Texas

**Table 2:** Demographics of primary dogs in a clinical field study investigating the safety and efficacy of Simparica Trio™

Category	Treatment group		
	Simparica Trio™ (*n* = 167)	Afoxolaner (*n* = 84)	Total (*n* = 251)
No. of females (%)	78 (46.7)	41 (48.8)	119 (47.4)
No. of males (%)	89 (53.3)	43 (51.2)	132 (52.6)
No. neutered (%)	99 (59.3)	60 (71.4)	159 (63.3)
No. not neutered (%)	68 (40.7)	24 (28.6)	92 (36.7)
Mean initial age in years (range)	5.1 (0.2–17.0)	4.9 (0.2–14.0)	5.0 (0.2–17.0)
Pure-bred/mixed breed %	50.3/49.7	48.8/50.2	49.8/50.2
No. short hair length (%)	91 (54.5)	50 (59.5)	141 (56.2)
No. medium hair length (%)	56 (33.5)	27 (32.1)	83 (33.1)
No. long hair length (%)	20 (12.0)	7 (8.3)	27 (10.8)
No. indoors and outdoors (%)	65 (38.9)	39 (46.4)	104 (41.4)
No. mostly indoors (%)	90 (53.9)	43 (51.2)	133 (53.0)
No. mostly outdoors (%)	12 (7.2)	2 (2.4)	14 (5.6)

Thirty dogs (24 in the combination group and six afoxolaner-treated) were withdrawn from the study prior to Day 60. The most common reason for withdrawal was owner-noncompliance with the protocol requirements (17 dogs). Five dogs were withdrawn at the discretion of the owner (e.g. owner no longer able or willing to participate in the study), two dogs due to the household being disqualified (addition/removal of dogs in the household), four dogs due to adverse events unrelated to treatment and two dogs went missing.

### Dose acceptance

Treatments were generally well accepted. For the combination product tablets, of a total of 517 doses, 91.9% were voluntarily accepted by free choice without, or when offered in food (74.5% without food, 17.4% in food); only 8.1% of doses had to be pilled. Of the 268 afoxolaner doses, 96.3% were voluntarily accepted by free choice or in food (89.2% without food, 7.1% in food); only 3.7% of doses had to be pilled.

### Efficacy

#### Flea counts

At the initial screening evaluation, primary dogs had flea counts ranging from 10 to 2,850 and mean counts were similar for dogs from the two treatment groups (Table 3). Both treatments significantly reduced the numbers of live fleas recovered at subsequent visits (23.78 ≤ *t*_(12)_ ≤ 36.51, *P* < 0.0001). The combination product produced efficacies based on geometric (arithmetic) means of 99.0% (98.5%) on Day 30 and 99.7% (99.7%) on Day 60, the respective efficacies for the afoxolaner-treated dogs were 98.3% (94.8%) and 99.6% (99.7%). The maximum number of live fleas recovered from any dog at Day 60 was eight fleas for the combination product group and four fleas from the afoxolaner group.

**Table 3:** Geometric (arithmetic) mean live flea counts, ranges and efficacies for dogs dosed orally with Simparica Trio™ or afoxolaner

Count day^a^	Simparica Trio™				Afoxolaner			
	*n*	Mean^b^	Range	% Efficacy	*n*	Mean^b^	Range	% Efficacy
0	149	42.5 (102.5)	10–2850	–	73	34.5 (86.1)	10–1220	–
30	142	0.4 (1.5)^c^	0–72	99.0 (98.5)	70	0.6 (4.5)^c^	0–148	98.3 (94.8)
60	136	0.1 (0.3)^c^	0–8	99.7 (99.7)	68	0.1 (0.3)^c^	0–4	99.6 (99.7)

^a^Day 0 = pre-treatment count; Day 30 = Days 26 to 35; Day 60 = Days 54 to 66

^b^Table displays the Day 0 geometric means from the Day 0 to Day 30 models. The Day 0 geometric means from the Day 0 to Day 60 model were 42.6 for the Simparica Trio™-treated group and 34.9 for the afoxolaner-treated group

^c^Mean flea count significantly lower than Day 0 (23.78 ≤ *t*_(12)_ ≤ 36.51, *P* < 0.0001)

#### Clinical signs associated with FAD

Due to prior corticosteroid use or other deviations that could confound interpretation of the clinical signs, a number of primary dogs were excluded from FAD evaluation. Clinical signs associated with FAD improved over the course of treatment in dogs in both treatment groups. Prior to the first treatment, 127 combination product-treated dogs were evaluable for FAD, these animals had clinical signs of pruritus (45.7%), papules (18.9%), erythema (35.4%), scaling (26.0%), alopecia from self-trauma (33.1%), and dermatitis/pyodermatitis (28.3%). Reductions in these clinical signs were seen within 30 days of the first treatment (Table 4). By Day 60, these signs had further improved with combination product-treated dogs showing markedly lower incidences of FAD clinical signs: pruritus (6.9%), papules (2.6%), erythema (7.8%), scaling (8.6%), alopecia from self-trauma (8.6%), and dermatitis/pyodermatitis (3.4%). Incidence and improvement in clinical signs of FAD was similar in the afoxolaner group (Table 4). Of those dogs with at least one clinical sign of FAD prior to the first study treatment, 94.3%, 90.0%, 87.2%, 79.3%, 86.8% and 90.3% of combination product-treated dogs, and 93.1%, 100%, 88.9%, 88.2%, 85.7% and 83.3% of afoxolaner-treated dogs showed a decrease in the severity of pruritus, papules, erythema, scaling, alopecia from self-trauma, and dermatitis/pyodermatitis, respectively, on Day 60 compared to pre-treatment.

**Table 4:** Percentage of dogs with clinical signs associated with flea allergy dermatitis dosed orally with Simparica Trio™ or afoxolaner

	Assessment day^a^	*n*	Pruritus	Papules	Erythema	Scaling	Alopecia from self-trauma	Dermatitis/Pyodermatitis
Simparica Trio™	0	127	45.7	18.9	35.4	26.0	33.1	28.3
	30	121	19.0	5.8	15.7	16.5	24.8	14.0
	60	116	6.9	2.6	7.8	8.6	8.6	3.4
Afoxolaner	0	66	47.0	21.2	31.8	28.8	24.2	31.8
	30	63	15.9	3.2	12.7	7.9	22.2	14.3
	60	61	6.6	1.6	6.6	6.6	14.8	4.9

^a^Day 0 = pre-treatment; Day 30 = Days 26 to 35; Day 60 = Days 54 to 66

### Health observations

The majority of the clinical signs observed and reported during the study were those typically observed secondary to flea infestation (consistent with allergies and dermatitis) or consistent with intermittent occurrences of conditions regularly observed in the non-study dog population and were similar in both treatment groups. Abnormal clinical signs occurring in > 2.0% of treated dogs in one or both groups included otitis externa, pruritus, diarrhea and emesis. Severe adverse events were reported for seven dogs during the study. In the combination product group, two dogs died after being hit by cars, one dog experienced an unrelated drug toxicity, one dog died from a ruptured spleen and another from aspiration pneumonia. In the afoxolaner group, one dog had peracute blindness and another had suspected diabetes mellitus. None of the severe adverse events that occurred in each treatment group were considered to be related to either treatment.

Mean body weights for the dogs in each treatment group were similar at Day 0 (combination product, 16.4 kg; afoxolaner, 17.3 kg) and dogs in both groups showed a tendency to slight weight increase (~ 1 kg increase in mean body weight for both groups by Day 60) during the study. Mean results for hematology and serum chemistry in both treatment groups were similar and within the normal respective reference ranges, the results of urinalyses also being unremarkable in both treatment groups.

Various concomitant medications and therapies were administered to dogs during this study and these were consistent with the demographics and study duration for the veterinary patient population examined. The most commonly administered (3% or greater for the total population) concomitant medications included those typically used in general veterinary practice: immunologicals, heartworm preventatives with or without a gastrointestinal dewormer, antibacterials, otologicals, other dermatological preparations, corticosteroids for systemic use, and anesthetic agents. Heartworm preventatives were used only by the afoxolaner-treated group since the control product did not provide heartworm prevention. All medications and therapies administered concurrently appeared to be well tolerated.

## Discussion

Dogs from a variety of regions in the contiguous USA, broadly representing the general dog population were included in this 2-month study to evaluate the safety and effectiveness of Simparica Trio™ for flea control under normal home use conditions. Simparica Trio^™^ dosed at 1.2–2.4 mg/kg sarolaner, 24–48 µg/kg moxidectin and 5–10 mg/kg pyrantel (as pamoate salt) was highly effective for the treatment and prevention of flea infestations. Prior to treatment, primary dogs included for efficacy assessment in the Simparica Trio™ group had geometric (arithmetic) mean flea counts of 42.5 (102.5) fleas per dog, with a maximum of 2850 fleas recovered from a single animal. Following the initial treatment on Day 0, mean live flea counts were reduced at Day 30 by 99.0% (98.5%) and at Day 60, after the second monthly treatment efficacy was 99.7% (99.7%). This level of effectiveness was similar to that attained by the commercial comparator product (afoxolaner).

The efficacy against fleas shown in this study is consistent with that demonstrated previously for sarolaner alone in a field study [18]. Additionally, reductions in the numbers of fleas seen in the post-treatment evaluations in these dogs in this study are consistent with the rapid onset of activity of Simparica Trio™ as demonstrated in a laboratory flea speed of kill study [19], with 100% reduction of flea egg-laying also being demonstrated in a laboratory flea reproduction study [19]. The combination of these effects results in the suppression of the flea populations in the dogs’ environments by killing newly emerged fleas on the dogs before they lay eggs and contribute to the environmental re-infestation [20]. This flea control is consistent with a high adulticidal activity and breaking of the flea life-cycle through the cessation of flea reproduction that was seen in laboratory studies [19].

Treatment with Simparica Trio™ resulted in the rapid resolution of the clinical signs associated with flea infestations and FAD. The majority of dogs with any clinical sign of FAD at study start showed marked improvement by the end of the study. Pruritus, the most common sign associated with flea infestation and FAD was observed in 45.7% of dogs on Day 0 and dropped to 6.9% at study conclusion.

The Simparica Trio™ chewable tablets were well accepted with the majority of offerings (91.9%) taken by free choice without food or in food and only 8.1% of tablets required pilling. This shows that the Simparica Trio™ chewable tablet formulation is palatable to most dogs and should be easy and convenient for owners to dose under normal use conditions.

Simparica Trio™ was well-tolerated and adverse events reported during the study were similar among dogs treated with both Simparica Trio™ and the commercial comparator treatment. The most common adverse events were associated with allergies and dermatitis, these most likely secondary to flea infestation (the target population) or with the intermittent occurrences of expected conditions in the non-study dog population. None of the severe adverse events that occurred in each treatment group were considered to be related to either treatment. In both treatment groups, clinical pathology findings were unremarkable, and the various concomitant medications such as vaccines, antibiotics, anesthetics and steroids that were administered concurrently during the study were well tolerated.

This field study confirmed the safety and effectiveness of monthly dosing with Simparica Trio™ for the treatment and prevention of fleas on dogs under normal home use conditions. This excellent flea control plus the added benefits of the combination providing coverage of the other major ecto- and endoparasites of dogs (ticks, heartworm, lungworm, gastrointestinal nematodes) in a single, palatable monthly dosage make this a convenient treatment option for veterinarians and dog owners [21–25].

## Conclusions

The novel combination product, Simparica Trio™, containing sarolaner, moxidectin and pyrantel, administered orally according to commercial directions once monthly for two consecutive treatments, was highly effective in the treatment and prevention of natural infestations of fleas on dogs presented as veterinary patients. Simparica Trio™ provided substantial improvement in clinical signs of FAD as the result of the rapid elimination of fleas. Simparica Trio™ tablets were readily accepted by free choice without or with food by most dogs. Simparica Trio™ was well tolerated by dogs even in combination with other common medications and therapies.

## Data Availability

The dataset supporting the conclusions of this article is included within the article.

## Abbreviations

FAD: flea allergy dermatitis
USA: United States of America
WAAVP: World Association for the Advancement of Veterinary Parasitology
